# Automatic Avoidance Tendencies for Alcohol Cues Predict Drinking After Detoxification Treatment in Alcohol Dependence

**DOI:** 10.1037/adb0000232

**Published:** 2016-12-01

**Authors:** Matt Field, Lisa Di Lemma, Paul Christiansen, Joanne Dickson

**Affiliations:** 1 Department of Psychological Sciences, University of Liverpool, and the UK Centre for Tobacco and Alcohol Studies, Liverpool, UK

**Keywords:** alcohol, ambivalence, approach, avoidance, implicit cognition

## Abstract

Alcohol dependence is characterized by conflict between approach and avoidance motivational orientations for alcohol that operate in automatic and controlled processes. This article describes the first study to investigate the predictive validity of these motivational orientations for relapse to drinking after discharge from alcohol detoxification treatment in alcohol-dependent patients. One hundred twenty alcohol-dependent patients who were nearing the end of inpatient detoxification treatment completed measures of self-reported (Approach and Avoidance of Alcohol Questionnaire; AAAQ) and automatic (modified Stimulus-Response Compatibility task) approach and avoidance motivational orientations for alcohol. Their drinking behavior was assessed via telephone follow-ups at 2, 4, and 6 months after discharge from treatment. Results indicated that, after controlling for the severity of alcohol dependence, strong automatic avoidance tendencies for alcohol cues were predictive of higher percentage of heavy drinking days (PHDD) at 4-month (β = 0.22, 95% CI [0.07, 0.43]) and 6-month (β = 0.22, 95% CI [0.01, 0.42]) follow-ups. We failed to replicate previous demonstrations of the predictive validity of approach subscales of the AAAQ for relapse to drinking, and there were no significant predictors of PHDD at 2-month follow-up. In conclusion, strong automatic avoidance tendencies predicted relapse to drinking after inpatient detoxification treatment, but automatic approach tendencies and self-reported approach and avoidance tendencies were not predictive in this study. Our results extend previous findings and help to resolve ambiguities with earlier studies that investigated the roles of automatic and controlled cognitive processes in recovery from alcohol dependence.

The decision to consume alcohol is determined by the balance between motivational inclinations to drink and inclinations to avoid drinking, hereafter referred to as “approach” and “avoidance” inclinations, respectively (Breiner, Stritzke, & Lang, 1999; McEvoy, Stritzke, French, Lang, & Ketterman, 2004). In alcohol-dependent patients, approach inclinations might arise from the desire for intoxication, whereas avoidance inclinations might arise from insight into the adverse consequences of chronic heavy drinking. Resolution of conflict between motivation to drink and motivation to abstain may be essential for the successful treatment of alcohol dependence and other addictions (Hettema, Steele, & Miller, 2005; Miller & Rollnick, 1991; Oser, McKellar, Moos, & Moos, 2010). According to dual-process theories of addiction (see Stacy & Wiers, 2010), these motivational inclinations may operate in both controlled (or “explicit”) and automatic (or “implicit”) cognitive processes. Controlled processes are rule-based, accessible to conscious awareness, and they are typically assessed with self-report measures. By contrast, automatic processes are associative, activated by environmental cues, and they are typically assessed with reaction time (RT) tasks. Controlled and automatic processes are thought to influence behavior independently, such that individual differences in automatic processes have a causal influence on behavior over and above that attributable to individual differences in controlled processes (Stacy & Wiers, 2010). In this article, we report findings from a prospective study in which we investigated the predictive validity of alcohol-related approach and avoidance inclinations, operating in controlled and automatic processes, for relapse to drinking in alcohol-dependent patients after detoxification treatment.

The Approach and Avoidance of Alcohol Questionnaire (AAAQ; McEvoy et al., 2004) measures the strength of self-reported approach and avoidance inclinations for alcohol. Factor analyses have confirmed that its Resolved-Regulated subscale, which captures inclinations to avoid drinking, is distinct from other subscales that capture inclinations to drink (Klein & Anker, 2013; Klein, Stasiewicz, Koutsky, Bradizza, & Coffey, 2007; McEvoy et al., 2004; Schlauch, Levitt, et al., 2013). That is, strong avoidance is not simply the inverse of weak approach, an observation that is supported by findings from laboratory studies in which approach and/or avoidance inclinations were experimentally dissociated (Curtin, Barnett, Colby, Rohsenow, & Monti, 2005; Di Lemma, Dickson, Jedras, Roefs, & Field, 2015; Jones, Rose, Cole, & Field, 2013; Schlauch, Breiner, Stasiewicz, Christensen, & Lang, 2013; Schlauch, Gwynn-Shapiro, Stasiewicz, Molnar, & Lang, 2013). Recent studies that used the AAAQ with alcohol-dependent patients demonstrated that approach and avoidance have differential predictive validity in this population: Strong avoidance (but not approach) predicts increased likelihood of entering into and engaging with treatment (Schlauch et al., 2012), whereas strong approach (but not avoidance) predicts a reduced likelihood of maintaining abstinence (Klein & Anker, 2013) and increased frequency of drinking and volume of alcohol consumed after discharge from treatment (Schlauch et al., 2012; see also Schlauch, Levitt, et al., 2013).

Regarding automatic processes, both appetitive (approach) and aversive (avoidance) alcohol-related processing biases are apparent in alcohol-dependent patients in a variety of domains, including attentional biases (e.g., Field, Mogg, Mann, Bennett, & Bradley, 2013), affective associations (e.g., Dickson, Gately, & Field, 2013), and approach and avoidance tendencies (Barkby, Dickson, Roper, & Field, 2012). The latter have been assessed with the alcohol-related Stimulus-Response Compatibility (SRC) task (Field, Kiernan, Eastwood, & Child, 2008) and related tasks (Wiers, Rinck, Dictus, & van den Wildenberg, 2009). In the standard version of the alcohol-related SRC task, participants are instructed to categorize alcohol-related and stationery-related (control) pictures by moving a manikin toward or away from the pictures. During an “approach alcohol” block of trials, participants move the manikin toward alcohol-related pictures and away from stationery-related pictures, whereas on a different “avoid alcohol” block, they do the opposite. Faster responding during “approach alcohol” blocks compared with “avoid alcohol” blocks is indicative of stronger alcohol-approach associations compared with alcohol-avoidance associations, whereas faster responding during “avoid alcohol” blocks indicates the opposite.

Dual-process theories (e.g., Stacy & Wiers, 2010) predict that chronic heavy drinking should lead to the development of strong associations between alcohol cues and behavioral approach; therefore, patients with alcohol dependence should be characterized by strong automatic approach tendencies for alcohol cues. However, studies that tested this prediction have yielded very inconsistent findings. One study demonstrated that alcohol-dependent patients were relatively faster to approach, rather than avoid, alcohol pictures, compared with approach and avoidance latencies for control pictures (Ernst et al., 2014). However, several other studies demonstrated the opposite, that is, relatively faster avoidance of alcohol than control pictures (Snelleman, Schoenmakers, & van de Mheen, 2015; Spruyt et al., 2013; Wiers, Eberl, Rinck, Becker, & Lindenmeyer, 2011), and others reported no difference in the speed of approach and avoidance of alcohol and control pictures (Barkby et al., 2012; Eberl et al., 2013). Two studies investigated the relationship between alcohol avoidance tendencies and relapse to drinking after treatment. The first demonstrated that patients with stronger avoidance tendencies were more likely to relapse to drinking 3 months after discharge from treatment (Spruyt et al., 2013). However, this effect was not replicated in a later study (Snelleman et al., 2015).

One explanation for the inconsistent findings in studies with alcohol-dependent patients is that the standard version of the alcohol-related SRC task yields an index of automatic approach tendencies that is *relative* to the strength of automatic avoidance tendencies (and vice versa). Given that alcohol-dependent patients report ambivalence about their drinking (Miller & Tonigan, 1996), it is plausible that alcohol cues may evoke strong automatic approach and avoidance at the same time. If this is correct, differences between studies in the strength of avoidance tendencies may partially account for inconsistent findings. We recently developed a modified version of the SRC task that is able to capture the strength of (a) automatic approach tendencies relative to neutral categorization responses, and (b) automatic avoidance tendencies also relative to neutral categorization responses (Baker, Dickson, & Field, 2014; Di Lemma et al., 2015). In recent studies that used this modified task, we demonstrated that heavy drinkers who were not seeking treatment were slower to avoid (Baker et al., 2014) or faster to approach (Di Lemma et al., 2015) alcohol cues compared with control cues. Importantly, no previous study has used this modified SRC task with alcohol-dependent patients to distinguish automatic approach and avoidance tendencies in this population.

In the present study, our primary aim was to investigate the predictive validity of alcohol-dependent patients’ approach and avoidance inclinations for alcohol that operate in both controlled and automatic processes. Several studies tested the predictive validity of the AAAQ, and other studies tested the predictive validity of the SRC task, but no previous study has combined these predictor variables in order to test predictions made by dual process theories (Stacy & Wiers, 2010)—namely, that variation in controlled processes (the AAAQ subscales, in this case) should predict variance in patients’ alcohol consumption after discharge from treatment, but variation in automatic processes (indices of automatic alcohol approach and avoidance tendencies) should predict additional variance in these outcomes. We hypothesized that, among alcohol-dependent patients enrolled in detoxification treatment, self-reported approach inclinations (as inferred from the AAAQ) would predict drinking outcomes after discharge from treatment, but self-reported avoidance inclinations would not be predictive. We also predicted that indices of automatic alcohol-related approach and avoidance tendencies (as inferred from the modified SRC task) would have incremental predictive validity beyond the variance that could be attributed to the AAAQ.

## Method

### Participants

One hundred twenty (71 males, 49 females; mean age = 43.45 years, *SD* = 8.88) alcohol-dependent inpatients were recruited from a specialist alcohol dependence treatment unit in Liverpool, United Kingdom. Alcohol dependence (ICD-10 criteria; World Health Organization, 1992) was diagnosed on the basis of structured clinical interviews. Patients were admitted for inpatient pharmacological detoxification and were discharged soon after withdrawal symptoms had subsided, typically after 1 week on the ward. Eligible participants were identified and approached by clinical nursing staff toward the end of detoxification when they were being considered for discharge, as they had recovered from symptoms of withdrawal. Inclusion criteria included fluency in English, normal or corrected-to-normal vision, and aged between 18 and 60 years. Exclusion criteria included psychosis, cognitive impairment, history of organic brain disease, and a current breath-alcohol concentration greater than zero. Ethical approval for the study was granted by the University of Liverpool, the National Research Ethics Committee, and the local National Health Service Trust. Individuals were given 24 hr to consider their decision to participate in the study, and all participants provided informed consent. Participant characteristics are reported in Table 1.[Table-anchor tbl1]

### Materials

#### The modified SRC task (Baker et al., 2014; Di Lemma et al., 2015)

The modified SRC task is a computerized task used to measure automatic approach and avoidance tendencies evoked by alcohol-related cues. Participants are instructed to rapidly categorize alcohol-related and stationery-related (control) pictures by moving a manikin either toward or away from the pictures, or to the left (neutral movement), as quickly as possible by pressing a specific key on the keyboard. This version of the task is a modification of the original version in which only approach and avoidance responses are required (see Field, Caren, Fernie, & De Houwer, 2011; Field et al., 2008; Field, Mogg, & Bradley, 2005; Kersbergen, Woud, & Field, 2015). As detailed below, the addition of movements to the side permits evaluation of the strength of approach and avoidance tendencies relative to a neutral movement rather than relative to each other. The task was programmed in Inquisit v3 software (Millisecond Software, 2006) and presented on a laptop computer with a 13-in. monitor screen.

The format of the task, trial structure, and perceptual characteristics of the pictorial stimuli were identical to those used in previous studies (Baker et al., 2014; Di Lemma et al., 2015). Fourteen colored pictures were used: seven pictures of alcoholic drinks and close-ups of individuals holding or consuming those drinks, and seven control pictures of stationery items and close-ups of models interacting with those items. These pictures were a subset of a larger picture set used in our previous study that used the standard SRC task with alcohol-dependent patients (Barkby et al., 2012).

On each trial of the task, a picture (alcohol-related or stationery-related) was presented in the center of the computer screen, with a manikin (matchstick man) presented either directly above or directly below the picture. Participants were instructed to move the manikin toward or away from the picture, or move it to the left, by pressing one of three keys on the keyboard labeled “up,” “down,” and “left.” There were four subblocks of the task, which differed according to task instructions. In the “approach alcohol” block, participants were required to move the manikin toward alcohol pictures, and to the left for stationery pictures. In the “avoid alcohol” block, participants moved away from alcohol pictures and to the left for stationary pictures. In the “approach control” block, participants moved toward stationery pictures and to the left for alcohol pictures. Finally, in the “avoid control” block, participants moved away from stationery pictures and to the left for alcohol pictures. Note that in the case of approach and avoidance movements, the position of the manikin was crucial: If the manikin was above the picture, an “approach” response required participants to press the “down” key, whereas an “avoidance” response required participants to press the “up” key; this was reversed if the manikin was below the picture.

Participants were instructed to respond as quickly and accurately as possible on each trial. If they pressed the correct key, the manikin moved up, down, or to the left in an animation lasting approximately 500 ms. If they pressed the wrong key, visual error feedback was presented for 500 ms. There was an intertrial interval of 500 ms. Each subblock of the task comprised four practice trials, in which two alcohol pictures and two control pictures were presented, once with the manikin above each picture type and once with the manikin below. If participants did not understand the task, this practice block was repeated. There then followed 28 “critical” trials, in which each of the 14 pictures was presented twice: once with the manikin above the picture, and once with the manikin below. Trials were presented in a new random order for each participant. Participants completed the subblocks in a counterbalanced order. Responses and RTs (in ms) to initiate the manikin movement were recorded on each trial.

#### AAAQ - Right Now version (McEvoy et al., 2004)

The AAAQ is a 14-item questionnaire that assesses motivational tendencies to approach or avoid drinking at that moment in time. Respondents are asked to rate how strongly they agree with each item on a nine-point Likert scale, from 0 (*not at all*) to 8 (*very strong*). Initial factor analysis of responses from nondependent drinkers (McEvoy et al., 2004) suggested a three-factor structure, with subscales labeled Inclined-Indulgent (mild approach, akin to desire to drink), Obsessed-Compelled (strong approach, akin to obsessive thoughts about drinking), and Resolved-Regulated (avoidance, or motivation to avoid drinking). However, subsequent factor analyses of responses from alcohol-dependent patients yielded a more inconsistent factor structure; all confirmed the independence of approach from avoidance motivation, but some studies suggested a single approach factor as opposed to the distinction between mild and strong approach (Klein et al., 2007; Schlauch, Levitt, et al., 2013), whereas others confirmed the three-factor structure as originally reported (Klein & Anker, 2013). We performed a principal components analysis on our own data and this yielded a three-factor structure that was similar to that reported by McEvoy et al. (2004), although it was notable that several “approach” items loaded on both the Inclined-Indulgent and Obsessed-Compelled factors originally identified McEvoy et al. Details of this Principal Components Analysis are available on request. Our analysis is based on the factor structure reported by McEvoy et al., and the internal reliabilities (Cronbach’s alpha) of each subscale in the present study were as follows: Inclined-Indulgent, α = .77, Obsessed-Compelled, α = .72, Resolved-Regulated, α = .82.

### Procedure

Before admission to the treatment unit, patients received a clinical assessment including diagnosis of alcohol dependence and other psychological disorders, a detailed drinking history including the Severity of Alcohol Dependence Questionnaire (SADQ; Stockwell, Hodgson, Edwards, Taylor, & Rankin, 1979; Stockwell, Murphy, & Hodgson, 1983), and completed a 1-month timeline followback drinking diary (Sobell & Sobell, 1992).

On the day of the testing, session participants provided a breath sample (all participants had a breath alcohol level of zero) before completing the SRC task, the AAAQ, and two additional self-report measures (see the online supplemental materials) in a fixed order. Participants then provided their contact details before being debriefed. The entire session, including rest breaks and debriefing, took no more than 50 min. Participants received a £10 (approximately $12 US dollars) High Street voucher to compensate them for their time. After discharge from detoxification treatment, patients were not required to return to the clinic for any follow-up treatment or clinical assessment. Therefore, our follow-up interviews were conducted by the researcher (who was not connected with the clinic) by telephone at 2, 4, and 6 months after the testing session. Participants were asked the following questions: (a) “Have you consumed any alcohol over the previous 2 months?”; (b) “If so, approximately how many days per week did you consume alcohol?”; (c) “On average how much alcohol was consumed on each day?”; and (d) “Have you had any additional contact with treatment services?” Telephone follow-ups are a feasible method for the monitoring of treatment outcome after residential treatment (Deane, Kelly, Crowe, Lyons, & Cridland, 2014), and, although inferior to in-person follow-ups (which were not possible for this study, as we were unable to offer participants an additional financial inducement to attend the clinic or university for follow-up visits), they are associated with superior participant retention compared with other follow-up methods such as e-mail (Johnson et al., 2015). To maximize retention, we called participants up to six times before coding them as study dropouts if we were unable to speak to them.

### Data Processing

Regarding the modified SRC task, the strength of automatic approach and avoidance tendencies for alcohol were calculated using the *D-*measure algorithm (Greenwald, Nosek, & Banaji, 2003). Full details are provided in Barkby et al. (2012), but, in essence, this involves computing a mean RT for each of the four subblocks after applying a penalty for trials on which errors are made. The average RT on each subblock considers all trials during the block, for example, both “approach alcohol” and “move to the left for stationery pictures” trials on the “approach alcohol” block. The difference between speed of responding on “approach alcohol” and “approach control” blocks provides an index of the strength of alcohol approach tendencies that is completely independent of the strength of alcohol avoidance tendencies; positive values are indicative of stronger alcohol approach tendencies. Similarly, the difference between speed of responding on “avoid alcohol” and “avoid control” blocks provides an index of the strength of alcohol avoidance tendencies that is completely independent of the strength of alcohol approach tendencies. We also analyzed SRC task data using more conventional methods (in which error penalties are not applied, and mean RTs are calculated for each subblock of trials; see Kersbergen et al., 2015, for explanation), but this did not change the outcome of the analyses reported here.

In order to identify predictors of drinking outcomes after discharge from detoxification treatment, our outcome measure was the percentage of heavy drinking days (PHDD) at each of the three follow-up assessments. This outcome measure is widely used in clinical trials of treatments for alcohol dependence, as it captures both the frequency and intensity of alcohol consumption (Fertig et al., 2012; Garbutt, Kampov-Polevoy, Gallop, Kalka-Juhl, & Flannery, 2010; Gual et al., 2013; Litten et al., 2013; Witkiewitz et al., 2014). We did not analyze drinks per drinking day because this variable was too skewed for analysis, and we do not report analyses of percent abstinent days because this variable was highly negative correlated with PHDD at each follow-up (*r*s > −.99 at each follow-up), which demonstrates that on almost all days on which participants consumed alcohol, they drank heavily. For this British sample, we defined PHDD as the percentage of days in which participants reported consuming in excess of eight (men) or six (women) units of alcohol, for which one unit equals 8 g of alcohol. This threshold corresponds to the definition of a “binge” for the purposes of government statistics on alcohol (Health and Social Care Information Centre, 2015), it is routinely used as an outcome measure in clinical trials in the United Kingdom (e.g., Crombie et al., 2014), and this volume (64 g/day for men, 48 g/day for women) is comparable with definitions of PHDD in other countries (Gual et al., 2013; Witkiewitz et al., 2014).

### Study Dropouts and Treatment of Missing Data

The total dropout rate was 46.7%: 25 participants (20.8%) dropped out of the study at 2-month follow-up, an additional four (3.4%) dropped out at 4-month follow-up, and an additional 27 (22.5%) dropped out at 6-month follow-up. Notably, variables related to participants’ alcohol use or problem severity at baseline (SADQ scores, or the quantity or frequency of alcohol consumption) were not associated with dropout at any of the follow-up points (*p*s > .05). In clinical trials, it is not recommended to assume that study dropouts have resumed heavy drinking because this yields biased estimates of the treatment effect (Hallgren & Witkiewitz, 2013). Instead, it is recommended to estimate missing data using either multiple imputation (MI) or full information maximum likelihood (Hallgren & Witkiewitz, 2013; Witkiewitz et al., 2014). Little’s missing completely at random (MCAR) test confirmed that our data were MCAR (*p* > .05); therefore, we used MI to estimate missing data. We used SPSS (Version 22) to produce a five iteration pooled estimate for each regression coefficient in the model (Hallgren & Witkiewitz, 2013); the *p* values and 95% confidence intervals reported are derived from these pooled estimates. Furthermore, *R*^2^ values were estimated from each iteration of the MI analysis: Each *R* coefficient was converted to Fisher’s *Z* before being combined then converted into *R*^2^ (see Harel, 2009).

### Data Analyses

We initially conducted within-subject *t* tests to compare the strength of participants’ self-reported approach relative to self-reported avoidance, and to compare the strength of automatic approach relative with automatic avoidance. We also compared both automatic alcohol approach and avoidance tendencies with zero using one-sample *t* tests in order to investigate whether the sample as a whole had robust automatic approach or avoidance tendencies for alcohol. Next, in order to identify associations between these constructs and participant characteristics at baseline, we performed Spearman’s rho correlations between these variables. To test our primary hypotheses, we used hierarchical regression analyses to identify variables that predicted PHDD at each follow-up (2, 4, and 6 months after discharge from treatment). We entered severity of alcohol dependence (SADQ scores) at baseline as the predictor in the first step of the regression models, before adding indices of self-reported and automatic approach and avoidance in the second step. In all analyses, we applied an alpha level of *p* < .05, with the exception of correlations between variables at baseline; here, given the large number of correlations that were conducted, we used a more conservative threshold for statistical significance (*p* < .01).

## Results

### Descriptive Statistics and Within-Subject Comparisons

The AAAQ indicated stronger self-reported avoidance of alcohol compared with approach: Scores on the Resolved-Regulated subscale were larger than scores on both the Obsessed-Compelled, *t*(119) = 9.55, *p* < .001, and Inclined-Indulgent, *t*(119) = 12.04, *p* < .001, subscales (see Table 1). On the SRC task, indices of automatic alcohol approach and automatic alcohol avoidance did not differ from each other, *t*(119) = 1.24, *p* = .22. In addition, neither value was significantly different from zero (one-sample *t* tests: *t*[119] = 1.65, *p* = .10, and *t*[119] = .10, *p* = .92, for approach and avoidance, respectively). Therefore, across the sample as a whole, participants were not faster to approach or avoid alcohol cues relative to control blocks of the task.

### Correlations Between Variables at Baseline

The volume of alcohol consumed was correlated with the severity of alcohol dependence (ρ = .42, *p* < .001). However, neither of these variables was associated with the AAAQ or SRC approach or avoidance indices (*p*s > .048). In addition, none of the AAAQ subscales were associated with SRC approach or avoidance indices (*p*s > .1).

### Relapse to Drinking and Percentage of Heavy Drinking Days (See Table 2)

Eighteen participants (15%) remained abstinent from alcohol for the entire 6-month follow-up period, whereas 46 (38%) relapsed to drinking within the follow-up period. Note that the percentage of abstainers decreased over time, whereas PHDD increased from the 2-month to the 4-month follow-up.[Table-anchor tbl2]

### Predictors of PHDD (See Table 3)

There were no significant predictors of PHDD at the 2-month follow-up, but the SRC avoidance index was a significant predictor of PHDD at both the 4- and 6-month follow-ups. There were no other significant predictors.[Table-anchor tbl3]

## Discussion

Among a sample of alcohol-dependent patients who were nearing the end of inpatient detoxification treatment, strong automatic alcohol avoidance tendencies predicted worse drinking outcomes (a higher PHDD) 4 and 6 months after discharge from detoxification treatment. However, self-reported approach and avoidance inclinations for alcohol did not predict drinking outcomes.

Our observation that strong automatic avoidance tendencies for alcohol cues was a significant predictor of drinking outcomes at 4 and 6 months after discharge from detoxification treatment, even after controlling for the severity of alcohol dependence at baseline, can be considered a replication of findings from an earlier study (Spruyt et al., 2013). There are several important differences between the present study and that reported by Spruyt et al. (2013): The earlier study used a standard version of the SRC task that is unable to distinguish between strong avoidance and weak approach, they used a dichotomous outcome variable (relapsed to dependent drinking, or not), did not report the dropout rate, included only one follow-up period (3 months after discharge from treatment), and their sample size (*N* = 40) was considerably lower than that in the present study (*N* = 120, of whom 53% were retained through the 6-month follow-up period). Despite these differences between studies, findings from both studies suggest that strong automatic alcohol avoidance tendencies are reliable predictors of poor drinking outcomes at 3 to 6 months after discharge from treatment. Our larger sample size combined with the use of a recommended outcome measure with appropriate treatment of missing values arising from study dropout (see Witkiewitz et al., 2014) suggest that the findings reported by Spruyt et al. are unlikely to be spurious. Furthermore, our modified SRC task (which we validated in previous studies with nondependent drinkers: Baker et al., 2014; Di Lemma et al., 2015) clarifies the nature of the earlier findings. Findings from the standard SRC task used by Spruyt et al. are ambiguous because they could be interpreted as strong avoidance, weak approach, or a combination of the two. In our study, we modified the SRC task in order to distinguish the strength of automatic approach and avoidance tendencies, and our findings demonstrate that it is strong avoidance rather than weak approach that is predictive of drinking after detoxification treatment. However, it is important to note that another recent study (Snelleman et al., 2015) observed no predictive relationship between the strength of automatic approach or avoidance (as assessed with a standard SRC task) and relapse to drinking (defined as a categorical variable) at 3-month follow-up in a sample of 59 participants. This highlights the need for further research to clarify the conditions under which strong automatic avoidance is predictive of relapse to drinking after treatment, and to delineate the magnitude and moderators of the effect.

Importantly, the observation that strong automatic avoidance tendencies are predictive of relapse to drinking after detoxification treatment does not imply that those tendencies play a causal role. For example, patients who have more negative experiences that are attributable to alcohol (e.g., interpersonal or health problems) would be expected to have stronger automatic avoidance tendencies evoked by alcohol cues, and patients with these characteristics would be those who are more likely to relapse to drinking after treatment (see Wiers, Gladwin, & Rinck, 2013). If this supposition is correct, automatic avoidance tendencies may be a marker of the underlying processes that influence behavior, rather than a direct determinant of behavior. These underlying processes might include ineffective engagement of coping responses when alcohol-related cues are encountered after discharge from treatment (see Niaura, Abrams, Demuth, Pinto, & Monti, 1989), although this speculation awaits empirical testing. It is also important to point out that our findings do not undermine recent demonstrations that strengthening alcohol-avoidance associations through cue avoidance training results in reduced risk of relapse to drinking after treatment in alcohol-dependent patients (Eberl et al., 2013; Manning et al., 2016; Wiers et al., 2011), although further research is required to reconcile these apparently conflicting observations.

We were unable to replicate recent findings that individual differences in the approach subscale(s) of the AAAQ predicted relapse to drinking after treatment, and there are several plausible explanations for this. First, mean scores on the approach subscales were noticeably lower in the present study compared with previous studies (Klein & Anker, 2013; Schlauch, Levitt, et al., 2013; Schlauch et al., 2012), which raises the possibility that AAAQ approach subscales may only have predictive validity once a minimum threshold has been exceeded. Second, and possibly related, participants in the present study completed the AAAQ only once, toward the end of detoxification treatment and shortly before discharge from the clinic. In some of the previous studies (Schlauch, Levitt, et al., 2013; Schlauch et al., 2012), participants had been in treatment for a considerably longer period of time before the AAAQ was administered (e.g., after 12 weeks of psychological therapy in Schlauch et al., 2012). Possibly, the predictive validity of AAAQ approach subscale(s) is most robust if approach and avoidance motivation are assessed after many months of psychological therapy. Finally, two of the previous studies demonstrated that changes in AAAQ subscales over time were predictive of subsequent changes in alcohol consumption (Schlauch, Levitt, et al., 2013; Schlauch et al., 2012); therefore, within-subject changes in approach and avoidance motivation, rather than their absolute values, may be most reliably predictive of individual differences in drinking behavior after treatment. Further research is required to disentangle these issues, and it is important to contrast the predictive validity of the AAAQ and measures of automatic alcohol approach tendencies when both are administered at multiple time points over the course of detoxification and psychological treatment for alcohol dependence.

Our study has limitations. First, our modified SRC task did not reveal reliable automatic tendencies to approach or avoid alcohol cues in our sample as a whole, because both *d* measures were not significantly different from zero. As noted in the introduction, some previous studies used different versions of SRC or related tasks and demonstrated that alcohol-dependent patients were faster to approach rather than avoid alcohol cues, whereas other studies demonstrated the opposite, and other studies demonstrated no difference. The validity of the modified SRC task (used in the present study) has been demonstrated in previous studies (Baker et al., 2014; Di Lemma et al., 2015), but further research is required to identify the task and sample characteristics that are necessary for detection of strong automatic approach or avoidance tendencies in alcohol-dependent patients. Second, we measured our variables of interest only once, but we know that both self-reported and automatic motivational orientations for alcohol change within individuals over time. For example, self-reported approach inclinations decline over time in alcohol-dependent patients who are seeking treatment (Schlauch, Levitt, et al., 2013), and automatic alcohol approach tendencies decline over time in adolescents (Janssen et al., 2015). Future studies of this type could measure these variables multiple times over the course of treatment in order to investigate the nature of change in these variables over the course of treatment, rather than the predictive validity of their absolute levels at one moment in time. Third, we were primarily concerned with individual differences in participants’ approach and avoidance motivation for drinking. Although these constructs clearly overlap with the construct of motivational ambivalence, we did not include a validated measure of participants’ ambivalence about drinking and their readiness to change, such as the The Stages of Change Readiness and Treatment Eagerness Scale (SOCRATES; Miller & Tonigan, 1996), which means that we were unable to replicate previous demonstrations that scores on the AAAQ and SOCRATES subscales tend to be highly correlated within alcohol-dependent populations (Schlauch et al., 2012). Finally, our study had a high dropout rate (21%, 24%, and 47% at the 2-month, 4-month, and 6-month follow-ups, respectively). Although high dropout is, unfortunately, the norm for prospective studies with alcohol-dependent patients (typical attrition rates range between 10% and 35%; Hallgren & Witkiewitz, 2013; Witkiewitz et al., 2014), future studies might maximize participant retention by conducting follow-up assessments in person rather than over the telephone, and by offering financial inducements for participants to attend these follow-up sessions. Our study also had strengths, including our robust approach to missing data (Witkiewitz et al., 2014), and it is the very first study to contrast the predictive validity of self-report and automatic measures of approach and avoidance motivational orientations for alcohol; therefore, it represents an important development beyond previous studies that measured these constructs in isolation (Klein & Anker, 2013; Schlauch, Levitt, et al., 2013; Schlauch et al., 2012; Snelleman et al., 2015; Spruyt et al., 2013).

In summary, we have demonstrated that strong automatic avoidance tendencies for alcohol are predictive of relapse to drinking after detoxification treatment, which replicates a previous finding while resolving some ambiguities with its interpretation. We were unable to replicate previous demonstrations that self-reported approach tendencies are also predictive of relapse to drinking after treatment, which could be attributable to our participants’ duration of abstinence and to the low strength of their approach tendencies at the time of assessment. Further research involving multiple assessments of both automatic and self-reported approach and avoidance tendencies for alcohol are required to extend these findings, and to clarify the relative importance of these automatic and controlled processes in long-term recovery from alcohol dependence.

## Supplementary Material

10.1037/adb0000232.supp

## Figures and Tables

**Table 1 tbl1:** Participant Characteristics and Predictor Variables

Variable	Value
Age (years)	43.45 ± 8.88
Gender ratio M:F (% male)	71:49 (59%)
Daily alcohol consumption (UK units)	33.64 ± 14.51
Weekly alcohol consumption (UK units)	227.96 ± 98.96
Severity of Alcohol Dependence Questionnaire	40.71 ± 8.27
AAAQ Inclined-Indulgent	1.69 ± 1.83
AAAQ Obsessed-Compelled	2.41 ± 2.06
AAAQ Resolved-Regulated	4.67 ± 2.64
SRC alcohol approach bias (*d* measure)	.07 ± .48
SRC alcohol avoidance bias (*d* measure)	−.00 ± .49
*Note*. Values are *M* ± *SD*. UK = United Kingdom; 1 unit = 8g alcohol; AAAQ = Approach and Avoidance of Alcohol Questionnaire; SRC = Stimulus-Response Compatibility Task.	

**Table 2 tbl2:** Number of Abstainers, Relapsers, and Study Dropouts, and Percentage of Heavy Drinking Days (PHDD) at Each Follow-Up

Variable	2 months	4 months	6 months
Abstainers *N* (%)	41 (34%)	32 (27%)	24 (20%)
Relapsers *N* (%)	54 (45%)	59 (49%)	40 (33%)
Dropouts *N* (%)	25 (21%)	29 (24%)	56 (47%)
PHDD *M* (*SD*)	38.60 (47.74)	50.82 (47.13)	47.43 (48.07)

**Table 3 tbl3:** Regression Analysis Investigating Predictive Validity of SADQ, AAAQ Subscales, and SRC Task D Measures for PHDD at 2-, 4-, and 6-Month Follow-Up Assessments

Variable	Cumulative		Simultaneous			
	Δ*R*^2^	Δ*F*^a^	β	*t*	*p*	95% CI
2-month follow-up						
SADQ	.001	.17	.05	.50	.621	[−.14, .24]
AAAQ Inclined-Indulgent	.05	1.20	.29	1.84	.069	[−.02, .61]
AAAQ Obsessed-Compelled			−.18	−1.09	.278	[−.52, .15]
AAAQ Resolved-Regulated			−.08	−.73	.465	[−.29, .13]
SRC alcohol approach bias (*d*)			.06	.54	.589	[−.16, .28]
SRC alcohol avoidance bias (*d*)			.13	1.19	.243	[−.09, .36]
4-month follow-up						
SADQ	.001	.10	.03	.34	.733	[−1.56, .22]
AAAQ Inclined-Indulgent	.06	1.52	.18	.91	.377	[−.24, .60]
AAAQ Obsessed-Compelled			−.12	−.63	.533	[−.51, .27]
AAAQ Resolved-Regulated			.04	.32	.747	[−.19, .26]
SRC alcohol approach bias (*d*)			.12	1.23	.221	[−.07, .32]
SRC alcohol avoidance bias (*d*)			.22	2.06	.043*	[.07, .43]
6-month follow-up						
SADQ	.001	.07	.01	.12	.902	[−.18, .21]
AAAQ Inclined-Indulgent	.06	1.50	.10	.44	.671	[−.43, .64]
AAAQ Obsessed-Compelled			−.02	−.10	.920	[−.47, .43]
AAAQ Resolved-Regulated			−.16	−.15	.884	[−.24, .20]
SRC alcohol approach bias (*d*)			.10	1.02	.309	[−.10, .31]
SRC alcohol avoidance bias (*d*)			.22	2.08	.039*	[.01, .42]
*Note*. There was no evidence of multicollinearity in any of the regression models, as all variance inflation factors were <2.6 for AAAQ measures and <1.4 for SRC measures. SADQ = Severity of Alcohol Dependence Questionnaire; AAAQ = Approach and Avoidance of Alcohol Questionnaire; SRC = Stimulus-Response Compatibility Task; PHDD = percentage of heavy drinking days; *df* = degrees of freedom.						
^a^ Step 1, *df* = (1,118); Step 2, *df* = (5,114).						
* *p* < .05.						
