# Supplementary material for: Automatic Avoidance Tendencies for Alcohol Cues Predict Drinking After Detoxification Treatment in Alcohol Dependence
Source: Psychol Addict Behav. 2016 Dec 1;31(2):171–9. doi: 10.1037/adb0000232 (PMC5343749; doi:10.1037/adb0000232)
Supplement: Supplementary file 1 [file ze6007163047so1.doc]

**Supplemental Materials**

**Automatic Avoidance Tendencies for Alcohol Cues Predict Drinking After Detoxification Treatment in Alcohol Dependence**

**by M. Field et al., 2016, *Psychology of Addictive Behaviors***

**http://dx.doi.org/10.1037/adb0000232**

Background

Our study had two secondary aims. First, we created an idiographic measure of participants’ approach and avoidance goals for alcohol, which was adapted from measures that have been used to investigate the importance of approach and avoidance goals in anxiety and depression (Dickson and MacLeod, 2004a, b, Dickson, Moberly, & Kinderman, 2011). This measure required participants to identify their approach goals (e.g., ‘to give me confidence in social settings’) and avoidance goals (e.g., ‘to avoid feeling hungover the next day’) in regard to their drinking and to rate each goal in terms of its importance and the likelihood that they would achieve that goal outcome in the future. This task yields rich information about the nature of approach and avoidance motivational orientations that operate in controlled processes, including the accessibility of those orientations (inferred from the number of approach and avoidance goals that participants can spontaneously generate), the importance attached to each type of goal, and the perceived likelihood of achieving each type of goal. We performed a secondary analysis to investigate the predictive validity of each of these indices for PHDD after participants had been discharged from treatment. Second, on the basis of claims that thought suppression plays an important role in addiction, particularly in relapse after treatment (Garland, Froeliger, & Howard, 2014; Moss, Erskine, Albery, Allen, & Georgiou, 2015), our participants completed a measure of trait thought suppression. This permitted us to test our secondary hypothesis that high levels of self-reported avoidance inclinations (the resolved-regulated subscale of the AAAQ) would be associated with elevated trait thought suppression (see Garland et al., 2014; Moss et al., 2015), which would in turn be associated with elevated automatic approach tendencies evoked by alcohol cues, (see Beadman, Das, Freeman, Scragg, West, & Kamboj, 2015; also Klein, 2007; Palfai, Monti, Colby, & Rohsenow. 1997).

Method

*Idiographic Drinking Goals task*. This instrument was modified from a version used to measure approach and avoidance goals in anxiety and depression (Dickson & MacLeod, 2004a, b). The version used in the present study required participants to list as many approach and avoidance goals as they could with respect to their future alcohol use, in response to the following open-ended prompts “*When thinking about my drinking, it will be important for me to...*” and “*When thinking about my drinking, it will be important for me to try to avoid...*” Participants were free to write down as many or as few goals as they could for each category (approach, avoidance), with 90 seconds allowed for each. In accordance with previous studies that used this task, we applied this time limit in order to control for variation in effort and motivation to complete the task. In order to familiarize participants with the task and to get them used to responding within a time limit, they initially completed a practice version in which they were asked to list as many words as they could that began with the letter ‘F’ within 90 seconds.

After participants had listed their alcohol-related approach and avoidance goals, they were asked to rate each goal on 7-point Likert scales assessing *Importance* of that goal (“H*ow important is it to you that you achieve this goal*”?) on a scale from 0 (not very important) to 0 (extremely important); and *Likelihood* “H*ow likely is that you will achieve this goal”?)* on a scale ranging from 0 (not very likely to happen) to 6 (extremely likely to happen).

*White Bear Suppression Inventory* (WBSI; (Wegner & Zanakos, 1994). The WBSI is a 15 item Likert-scale questionnaire that measures tendencies to engage in thought suppression. Participants rate their agreement with statements a 5 point Likert scale ranging from 1 (strongly disagree) to 5 (strongly agree). Higher scores of the WBSI evidence greater tendencies to engage in thought suppression.

Results

See Table S1 for descriptive statistics. Participants generated more approach than avoidance goals (t(119) = 3.31, p < .001), and gave higher likelihood ratings to approach than avoidance goals, meaning that they perceived themselves as more likely to achieve approach than avoidance goals (t(119) = 7.44, p < .001). However, approach and avoidance goals were rated as equally important (t(119) = .56, p = .58). None of the idiographic goal measures were significant predictors of PHDD at any of the follow-up periods (see Table S2).

Correlations: Outcome measures from the idiographic goal task were unrelated to any of the other measures (ps > .015), with the exception of a significant positive correlation between the number of approach goals generated, and scores on the WBSI (rho = .26, p = .004). Importantly, correlations between thought suppression (WBSI) and the resolved-regulated subscale of the AAAQ (rho = .05, p = .60) and both indices from the SRC task (rho = .10, p = .27 and rho = -.02, p = .84 for SRC approach and avoidance, respectively) were all non-significant. This means that we reject our hypothesis regarding an indirect link between scores on the AAAQ resolved-regulated subscale and SRC automatic approach tendencies that is mediated by thought suppression.

Discussion

Idiographic measures of participants’ approach and avoidance alcohol-related goals did not predict relapse to drinking after they had been discharged from treatment. Furthermore, neither idiographic alcohol-related goals or individual differences in thought suppression were associated with our primary variables (automatic and self-reported approach and avoidance motivational orientations for alcohol) in cross-sectional analyses.

Regarding the idiographic goals task, we adapted a task that we have previously used to investigate the nature of approach and avoidance goals in patients with anxiety and depression (Dickson & MacLeod, 2004a, b, Dickson et al., 2011), and the present study was the first to adapt this task for alcohol-dependent patients. We found that participants generated more approach-related than avoidance-related goals, and they believed that they were more likely to achieve their approach-related goal outcomes rather than their avoidance-related goal outcomes in the future. However, individual differences in the number of approach and avoidance goals generated, and their rated importance and likelihood, were not predictive of relapse to drinking. Ours is not the first study to attempt to measure alcohol-related goals ideographically (see Klinger & Cox, 1986, Lecci*,* MacLean, & Croteau, 2002). Most relevant here, Klinger and Cox (1986) demonstrated that sustained abstinence after treatment for alcohol dependence was related to lack of concerns about avoiding alcohol, and optimism about achieving abstinence goals, a pattern of findings that appears inconsistent with our own. However, this study did not explicitly partition alcohol-related goals into approach-related and avoidance-related goals, so caution is required before making direct comparisons across studies. We suggest that further work is required to develop and validate a task for the idiographic assessment of participants approach and avoidance alcohol goals, perhaps initially in the form of cross-sectional studies that attempt to maximize its convergent validity with other measures of approach and avoidance goals, such as the AAAQ.

Contrary to hypotheses, we observed no statistically significant associations between individual differences in trait thought suppression and any of our measures of automatic or self-reported approach and avoidance tendencies for alcohol. These data do not support recent claims that strong motivational inclinations to avoid alcohol will prompt increased suppression of unwanted thoughts (e.g. craving), which will in turn increase the risk of relapse to drinking after treatment (Garland et al., 2014, Moss et al., 2015). Our findings are also incompatible with previous demonstrations that elevated thought suppression is associated with increased accessibility of automatic alcohol-related cognitions (Klein, 2007, Palfai et al., 1997), although it is notable that neither of these earlier studies measured automatic approach or avoidance tendencies using a modified SRC task as in the present study. Nonetheless, we suggest that one avenue for future research would be to develop and validate a measure of thought suppression that is specific to alcohol-related thoughts (rather than the more generalized measure of trait thought suppression that was used in many of the existing studies, as well as in the current study), because one might expect this to have greater convergent validity with measures of motivation to avoid alcohol, and predictive validity for relapse to drinking after treatment.

Table S1: Descriptive statistics for the idiographic goals task. Values are mean ± standard deviation

♯ alcohol-related approach goals generated 6.33 ± 2.77

♯ alcohol-related avoidance goals generated 5.42 ± 2.69

Approach goal importance (1-7) 6.56 ± 0.83

Avoidance goal importance (1-7) 6.62 ± 0.75

Approach goal likelihood (1-7) 5.91 ± 1.09

Avoidance goal likelihood (1-7) 4.31 ± 2.03

Table S2: Regression analysis investigating predictive validity of SADQ and idiographic drinking goals measures for PHDD at two-, four- and six-month follow-up assessments

Cumulative Simultaneous

Variable R^2^ change F-change^†^ *β* *t* p 95% CI

Two-month follow-up

SADQ .001 .17 0.04 0.37 .715 -0.17 to 0.25

Number of approach goals .028 1.20 0.08 0.71 .478 -0.14 to 0.29

Approach goal importance 0.02 0.11 .914 -0.27 to 0.31

Approach goal likelihood -0.04 -0.26 .796 -0.31 to 0.24

Number of avoidance goals -0.20 -1.81 .071 -0.41 to 0.02

Avoidance goal importance -0.02 -0.15 .879 -0.21 to 0.18

Avoidance goal likelihood -0.01 -0.02 .981 -0.22 to 0.22

Four-month follow-up

SADQ .001 .10 0.03 0.29 .775 -0.18 to 0.24

Number of approach goals .028 1.52 -0.12 -0.98 .330 -0.35 to 0.12

Approach goal importance -0.05 -0.34 .736 -0.35 to 0.25

Approach goal likelihood -0.02 -0.15 .881 -0.33 to 0.28

Number of avoidance goals 0.07 0.55 .585 -0.17 to 0.30

Avoidance goal importance 0.02 0.21 .832 -0.18 to 0.22

Avoidance goal likelihood -0.04 -0.26 .803 -0.41 to 0.32

Six-month follow-up

SADQ .001 .07 0.01 0.03 .979 -0.20 to 0.21

Number of approach goals .051 1.50 -0.18 -1.26 .225 -0.47 to 0.12

Approach goal importance 0.08 0.54 .591 -0.21 to 0.36

Approach goal likelihood -0.14 -0.94 .353 -0.44 to 0.16

Number of avoidance goals 0.04 0.34 .737 -0.17 to 0.24

Avoidance goal importance 0.05 0.53 .595 -0.14 to 0.25

Avoidance goal likelihood 0.05 0.39 .701 -0.23 to 0.33

^†^Step one df = (1,118); Step two df = (6,113)
